# Comparison between a Flash Glucose Monitoring System and a Portable Blood Glucose Meter for Monitoring of Cats with Diabetic Ketosis or Ketoacidosis

**DOI:** 10.3390/ani14192787

**Published:** 2024-09-26

**Authors:** Jennifer S. Eiermann, Katarina Hazuchova, Beatriz Vidondo, Miguel Campos, Simone Schuller, Yi Cui

**Affiliations:** 1 Division of Small Animal Internal Medicine, Department of Clinical Veterinary Science, Vetsuisse Faculty, University of Bern, 3012 Bern, Switzerland; jennifer.eiermann@unibe.ch (J.S.E.); simone.schuller@unibe.ch (S.S.); 2 Department of Veterinary Clinical Sciences, Small Animal Clinic, Internal Medicine, Justus-Liebig University Giessen, 35392 Giessen, Germany; katarina.hazuchova@vetmed.uni-giessen.de; 3 Veterinary Public Health Institute, Vetsuisse Faculty, University of Bern, 3012 Bern, Switzerland; beatriz.vidondo@unibe.ch; 4 Medisch Centrum voor Dieren, 1014 AS Amsterdam, The Netherlands; miguel.campos@anicura.nl

**Keywords:** FreeStyle Libre 2.0, AlphaTRAK 2, feline diabetes

## Abstract

**Simple Summary:**

Diabetic ketosis and ketoacidosis are severe and potentially life-threatening complications of diabetes mellitus in cats. Affected patients require intensive glucose monitoring. This monitoring is most commonly performed by using handheld portable blood glucose meters that require a sample of capillary blood. Glucose monitoring can be stressful for patients and veterinary personnel. A novel continuous flash glucose monitoring system (FreeStyle Libre 2.0 Abbott^®^) eliminates the need for frequent capillary blood sampling. In this study, however, the latest FreeStyle Libre 2.0 Abbott^®^ measured significantly lower glucose concentrations than the handheld blood glucose meter, both in well-hydrated and dehydrated cats. False low measurements do not lead to insulin overdosing, which can be detrimental to the patient, but may nonetheless lead to suboptimal insulin management. Additionally, the agreement between the FreeStyle Libre 2.0 Abbott^®^ and a handheld device varied significantly between cats.

**Abstract:**

Cats with diabetic ketosis or ketoacidosis DK(A) require intensive glucose monitoring. The aim of this study was to assess the agreement between a portable blood glucose meter (PBGM) and a flash glucose monitoring system (FGMS; FreeStyle Libre 2.0 Abbott^®^) measuring interstitial glucose in cats with DK(A). Ten client-owned cats with naturally occurring DK(A) were prospectively enrolled. Glucose concentrations were assessed with both methods every 1–3 h until resolution of DK(A), and every 4–8 h thereafter. While the median FGMS measured glucose concentration (14.3 mmol/L) was significantly lower than the median PBGM measured glucose concentration (19 mmol/L) (*p* < 0.001), the overall correlation between the FGMS and PBGM was high (r = 0.88; *p* < 0.001). In the Parkes error grid analysis, 98.3% of measurements fell in zones A and B. Bland–Altman plot analysis demonstrated that in the low glycaemic range (BG < 5.5 mmol/L), 50% of FGMS measurements deviated more than ±0.83 mmol/L, and in the high glycaemic range (BG > 5.5 mmol/L), 81% of results deviated >15% from the PBGM measurements. There was significant inter-individual variation in the difference between glucose concentrations measured by the FGMS and PBGM (*p* < 0.001). In spite of being more easily tolerated and easier to use, currently this method cannot be recommended for routine monitoring of cats with DK(A).

## 1. Introduction

Diabetic ketoacidosis (DKA) is a life-threatening complication of diabetes mellitus (DM). Insufficient insulin secretion by pancreatic beta cells leads to a relative or absolute insulin deficiency, which, in combination with counterregulation by stress hormones, can lead to a state of metabolic derangement characterised by severe hyperglycaemia and accumulation of ketone bodies [1]. The generation of ketones is linked to the release of hydrogen ions, which can overwhelm the body’s buffering systems, leading to metabolic acidosis [2]. Treatment consists of intravenous fluid administration, correction of electrolyte and acid-base abnormalities, followed by insulin administration and concurrent glucose supplementation when needed [2,3]. Typical complications include hypophosphataemia, hypokalaemia, anaemia, and hypoglycaemia [2,3]. Frequent glucose monitoring is necessary to adjust the dose of insulin and the rate of glucose supplementation [2,3]. Currently, monitoring of blood glucose (BG) concentration is most frequently performed using portable BG meters (PBGMs) from venous or capillary blood [4]. However, frequent blood sampling is stressful for the feline patient and can potentially lead to hospital-acquired anaemia [5].

In the past decade, less invasive continuous glucose monitoring (CGM) systems have been introduced in veterinary medicine. With these, glucose concentration is measured in the subcutaneous interstitial fluid using transcutaneous sensors. Interstitial glucose (IG) equilibrates with BG with a delay of 11–30 min in cats [4,6], with multiple studies showing a good correlation between these two compartments [6,7,8,9,10,11,12,13,14]. Similar findings have been described in human patients [15]. Difference in the capillary blood glucose concentrations measured at different anatomical sites (e.g., fingertip vs. forearm) have also been described [16]. Several CGM systems used in human medicine have also been validated in veterinary patients [7,8,9,10,11,12]. These devices must be regularly calibrated with blood samples to obtain reliable measurements.

Recently, a novel flash glucose monitoring system (FGMS) (FreeStyle Libre 2.0 Abbott^®^, Abbott Laboratories, Maidenhead, Berkshire, UK), later referred in the text as the FGMS 2.0, has been licensed for use in humans. This new system has two main advantages: it is inexpensive and is factory calibrated, meaning that no additional blood sampling for external calibration is required. It contains a transcutaneous sensor that measures IG every minute and automatically stores the values at 15 min intervals over a period of 8 h. The current IG value is displayed whenever the sensor is scanned with a dedicated reader or a smartphone app. At the same time, the continuous IG values of the previous 8 h are uploaded to the cloud storage.

The first generation of the FGMS has recently been validated for use in diabetic dogs, and in dogs with DKA [4,13,14]. The FGMS showed good clinical accuracy in canine patients compared to PBGM measurements, although a significant inter-individual variation in accuracy was identified in dogs with DKA [4,14]. In cats, good clinical accuracy has also been demonstrated but with a substantial individual variation in accuracy and discordance between IG and BG during periods of rapid change [6,17,18,19]. Factors such as application site or skin thickness have been shown to influence the accuracy of IG measurements and contribute to individual variations in both dogs and cats [19,20,21]. According to the manufacturer’s website, the second generation FGMS (FGMS 2.0), with improved calibration, is reported to be more accurate in people and less prone to inter-individual variation than the original FGMS (FreeStyle Libre).

The overall aim of this study was to evaluate the utility of the FGMS 2.0 as a novel glucose monitoring system in hospitalised cats with diabetic keto(acid)osis (DK(A)). The specific goals of this study were (1) to assess the agreement between a portable blood glucose meter (PBGM; AlphaTRAK 2, Zoetis, Parsippany, NJ, USA) and a flash glucose monitoring system (FGMS; FreeStyle Libre 2.0 Abbott^®^) including the evaluation of a potential inter-individual variation; (2) to evaluate the influence of hydration on the agreement between the two devices; and (3) to evaluate owners’ satisfaction with the FGMS 2.0 at home.

## 2. Materials and Methods

### 2.1. Study Design

Client-owned cats with naturally occurring DK(A) admitted to the Small Animal Clinic of the University of Bern and the University of Giessen between October 2020 and April 2022 were enrolled into this prospective, observational study. As the study did not lead to any deviation from the routine diagnostic and patient care protocols for cats with DK(A), the study did not qualify as an animal experiment according to the Swiss federal animal welfare legislation and was exempt from formal ethical approval. All owners signed an informed consent form upon enrolment. As an incentive, the sensor was provided and placed free of charge.

### 2.2. Cats

A diagnosis of DK was based on compatible clinical signs (e.g., polyuria, polydipsia, anorexia, vomiting, dehydration, apathy) and the presence of hyperglycaemia (BG ≥ 15 mmol/L), glucosuria, ketonaemia (beta-hydroxybutyrate ≥ 2.5 mmol/L) and/or ketonuria. In case of DKA, an increased anion gap metabolic acidosis needed to be present (pH < 7.34, bicarbonate ≤ 20 mmol/L, anion gap ≥ 21 mmol/L).

Cats suffering from DKA were treated following a protocol consisting of intravenous fluid therapy, electrolytes, and phosphate supplementation as needed, and intravenous (IV) continuous rate infusion (CRI) of short-acting regular insulin (Actrapid^®^, Novo Nordisk, Mainz, Germany) adjusted to the patients’ glycaemia [22]. Intravenous glucose supplementation was provided when needed based on BG measurements. The patients suffering from diabetic ketosis that retained a good appetite and general condition were treated with symptomatic fluid therapy and intermittent intermediate SC insulin. Depending on the patient’s clinical status, BG and IG concentrations were simultaneously assessed every 1–3 h until the CRI insulin was switched to an SC intermittent insulin regimen, and every 4–8 h thereafter until discharge using PBGM. It was decided to allow for sampling to be performed within these time limits to minimize stress for patients and to still allow increased monitoring frequency in patients with more severe disease, at the discretion of the attending veterinarian.

### 2.3. Flash Glucose Monitoring System Placement

The FGMS 2.0 was placed on the day of admission if the cat was presented during working hours or the following day if it was presented out-of-hours.

This system measures glucose concentration by using a glucose oxidase enzyme that allows glucose to oxidize and transfer electrons to a metal electrode, which produces a current. The glucose present in the interstitial fluid is proportional to the strength of the current produced [23].

Placement was performed as previously described [6]. Briefly, the dorsal cervical area was clipped and cleaned with alcohol wipes. Instead of tissue glue, Lash glue (Jolifin Lashes—Wimpernkleber Sensitiv, SAB Store GmbH & Co, Babenhausen, Germany) was applied to the sensor to improve adherence to the skin. This change was made because of previously reported incidents of skin reactions presumably caused by the surgical tissue glue [6]. After the sensor application, additional protective tape (Hypafix, BSN medical GmbH, Hamburg, Germany) and a small bandage (Rolta soft and Dermaplast CoFix, Hartmann, Heidenheim an der Brenz, Germany) were applied around the neck. Once applied, the sensor was scanned and after an initiation period of 1 h, IG values were recorded continuously.

### 2.4. Data Collection

Clinical parameters: The body condition score (BCS) was recorded upon admission using a previously described 9-point scoring system (World Small Animal Veterinary Association Global Nutrition Committee, BCS chart) [24]. The patient’s hydration status and body weight were assessed three and two times daily, respectively. Hydration was evaluated based on skin turgor, moisture of mucus membranes and alteration of the body weight. As veterinarians at different levels of training were assessing hydration status, euhydration was confirmed by a diplomate of the European/American College of Veterinary Internal Medicine or the European/American College of Veterinary Emergency and Critical Care. Hydration was assessed three times daily when a glucose measurement was performed, but not at every glucose assessment, to avoid additional stress for the patients. If the sensor stopped working or fell off during hospitalisation or at home, it was removed and the application site or a photo of it sent by the owner inspected for any signs of irritation.

Glucose concentrations: The BG concentration was recorded every 1–3 h until the switch from insulin CRI to an SC intermittent insulin regimen, and every 4–8 h thereafter until discharge using PBGM. The IG value from the FGMS 2.0 was obtained at the same time point. To allow for sampling at the same time, the pin prick was performed, and the glucose concentration was measured with the AlphaTRAK 2. As soon as a value was obtained, the FGMS sensor was scanned, and the IG glucose concentration was recorded. According to the manufacturers, both devices have specific ranges within which they can reliably detect glucose concentrations (FGMS 2.0 sensor: 1.1–27.8 mmol/L; AlphaTRAK 2: 1.1–41.7 mmol/L). Both devices might, however, show numeric readings that lie outside of this range or show “LO” or “HI”. For statistical analysis, whenever the FGMS 2.0 or the PBGM measured a glucose concentration below or above the detection limit (“LO” or “HI”), the value that was assigned and recorded was the threshold of the device + 0.1 mmol/L in case of “HI” or −0.1 mmol/L in case of “LO”, respectively.

Questionnaire: If cats were discharged with the sensor still in place, owners were asked to complete a questionnaire to assess their satisfaction with the FGMS 2.0 at home (see Supplementary Materials).

### 2.5. Statistical Analysis

Statistical analysis was performed using commercially available software (NCSS, 2021). NCSS: Statistical Software. NCSS LLC, Kaysville, UT, USA; R Core Team (2021). R: A language and environment for statistical computing. R Foundation for Statistical Computing, Vienna, Austria (v 4.1.2; R Core Team 2021).

The mean and standard deviation (SD) of the absolute difference between IG and BG were calculated, resulting in a mean of 5.6 mmol/L and an SD of 4.8 mmol/L. A power analysis was performed and revealed that a sample size of nine animals provides 80% power to detect a significant difference from zero in the mean of the paired differences, i.e., a two-sided *p*-value < 0.05 [25].

Data distributions were checked for normality by examining histograms and by the Shapiro–Wilk test. Given the non-normal distribution of most variables, the data are pre-sented as medians (range). The absolute difference between IG and BG was calculated as IG–BG; relative difference between IG and BG was calculated as ((IG − BG)/IG)*100. Kruskal–Wallis ANOVA (KWA) was used to evaluate differences in absolute and relative difference depending on the hydration state. To test for differences between median IG and BG, the Wilcoxon signed-ranks test was used.

Inter-individual variability was assessed by drawing box plots for each patient and performing a Kruskal–Wallis analysis (KWA) to compare the median differences between IG and BG measurements among cats. The statistical significance level was set at *p* < 0.05. A post hoc analysis was performed to determine which specific cats exhibited significant differences. To adjust the significance level for multiple comparisons, the Bonferroni correction was applied. The correlation between glucose concentrations measured by the FGMS and PBGM was analysed using Pearson’s correlation.

For the purpose of comparability to other studies evaluating CGMS/FGMS devices [4,6,13,17,18,26,27,28,29], ISO 15197:2013 criteria [30,31] were used to assess the agreement between FGMS and PBGM, although these two methods measure glucose in different compartments (interstitial vs. blood glucose). These criteria were developed to standardise the validation of a novel PBGM for stable diabetic humans and are commonly applied in veterinary studies. The following parameters were assessed: mean absolute relative difference (MARD), median absolute relative difference (mARD), mean relative difference (MRD), and mean absolute difference (MAD), as well as Bland–Altman plot to assess analytical accuracy and Parkes error grid analysis (EGA) to assess clinical accuracy [29]. MARD and mARD measure the absolute difference but not the direction (under- or overestimation of the FGMS) as a percentage. MRD measures the size and direction of the difference between FGMS and PBGM as a percentage. MAD reports the absolute difference and not a percentage.

The Parkes consensus plot, an error grid analysis (EGA), represents the percentage of values within different zones [29]. The zones A–E represent the risk related to possible inaccuracies in measurements in stable diabetic humans: no effect on clinical action (A), altered clinical action—little or no effect on clinical outcome (B), altered clinical action—likely to affect clinical outcome (C), altered clinical action—could have significant medical risk (D), altered clinical action—could have dangerous consequences (E).

In the Bland–Altman plot, for good analytical accuracy, based on ISO 15197:2013 criteria, at least 95% of all observations must be within ±0.83 mmol/L of the BG concentration for BG concentrations < 5.5 mmol/L (low glucose concentration) and within ±15% of the BG concentration for BG concentrations ≥ 5.5 mmol/L (high glucose concentration). In the EGA, for a good clinical accuracy, 99% of all glucose measurements must fall within zones A and B.

## 3. Results

### 3.1. Cats

**Figure 1:**
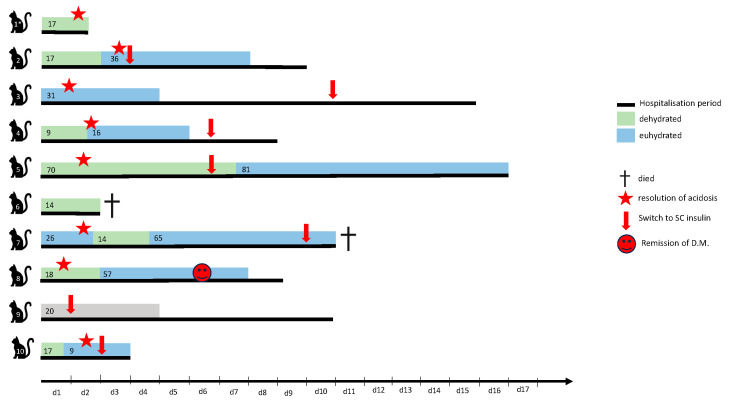
Overview of the duration of hospitalisation for each individual cat. Hospitalisation time and number of paired measurements during de- or euhydrated state are shown. Time to switch from insulin continuous rate infusion to intermittent intermediate SC insulin is depicted by the red flash, resolution of acidosis is depicted by the red star. For cat 9, clinical information on hydration status and resolution of acidosis was not available. This cat was excluded from all analyses relating to hydration status but was included in the overall analysis.

Ten cats were included in the study. Cats were between one and 13 years old (median 9; IQR 6.5–10). Seven cats were castrated males, and three were spayed females. There were seven Domestic Shorthair, two British Shorthair and one Maine Coon cat. The median BCS was 5/9 (IQR 3–5.5; range 2–9/9). In one cat, BCS was not recorded. Median time to switch from insulin CRI to intermittent intermediate SC insulin was 5 days (IQR: 2.75–6.75; range 1–10). One cat went into remission during hospitalisation and therefore no €C insulin administration was started after stopping insulin CRI. Two of 10 cats died during hospitalisation. Eight cats were discharged after a median hospitalisation period of 9.5 days (IQR 6.75–11.25; range 2–16) (Figure 1). For one cat, clinical information on hydration status and resolution of acidosis was not available. This cat was excluded from all analyses relating to hydration status but was included in the overall analysis. Additional information depicted in Figure 1 is discussed further in the Section 3.

### 3.2. Flash Glucose Monitoring System Placement

Application of the FGMS 2.0 was easy, well tolerated and did not cause any adverse reactions. The sensor was in place allowing glucose measurement for a median of 5.5 days (IQR 3–8.5; range 2–16 days). In all but one cat the sensor failed before the 14-day runtime period described by the manufacturer. In none of the cats was the sensor removed or displaced by the cat itself during hospitalisation.

No sensor-relevant adverse effects such as discomfort, scratching or signs of pain were recorded during hospitalisation. After removal of the sensor, no skin abnormalities such as erythema, crust formation or skin ulcerations were noted. New hair growth continued normally after removal of the sensor.

### 3.3. Agreement of the FGMS 2.0 and PBGM

A total of 517 paired BG and IG measurements with a median of 28.5 paired measurements per cat (IQR 19.3–82.5; range 14–151) were analysed. The number of measurements per cat and duration of glucose monitoring varied depending on the cats’ clinical status and their tolerance of blood sampling (Figure 1); 4.6% of the BG (24/517) and 7.5% of the IG (39/517) measurements were outside of the reliable measuring range indicated by the manufacturer. In 2.9% of the FGMS and 1.9% of the PBGM measurements, the systems indicated “HI” and the upper limit of the reference range +0.1 mmol/L was used as final concentration. The system never indicated “LO” in this study. The remaining concentrations lying outside of the reliable measuring range were shown as numeric values. Exclusion of all measurements outside the reliable measuring range from the statistical analysis did not alter the overall results. However, since measurements outside the reliable measuring range might still be used for clinical decision-making, the results of all collected data points, including those outside this range, are presented.

**Figure 2:**
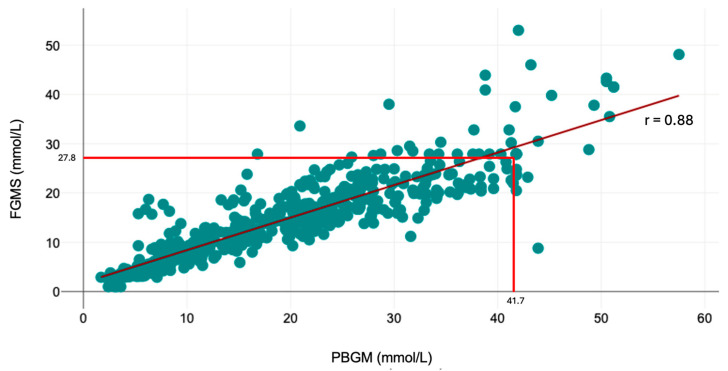
Scatter plot depicting agreement between IG measured by the FGMS and BG measured by the PBGM. Red lines mark the upper limit of the reliable measuring ranges for the PBGM and FGMS, respectively, as indicated by the manufacturers. Linear regression (calculated as y = 0.6596x + 1.8181) is indicated as the dark red line. Correlation coefficient is indicated as r.

There was a good overall correlation between the FGMS and PBGM (correlation coefficient r = 0.88, *p* < 0.001). The scatter plot (Figure 2) shows a linear trend for the FGMS to render lower values than the PBGM. The median glucose concentration measured by the FGMS 2.0 was significantly lower than the glucose concentration measured by the PBGM (14.3 mmol/L, IQR, range, 1.1–53 mmol/L vs. 19 mmol/L, IQR, range, 1.7–57.5 mmol/L; *p* < 0.001).

### 3.4. Overall Analytical and Clinical Accuracy Based on ISO 15197:2013 Criteria

**Figure 3:**
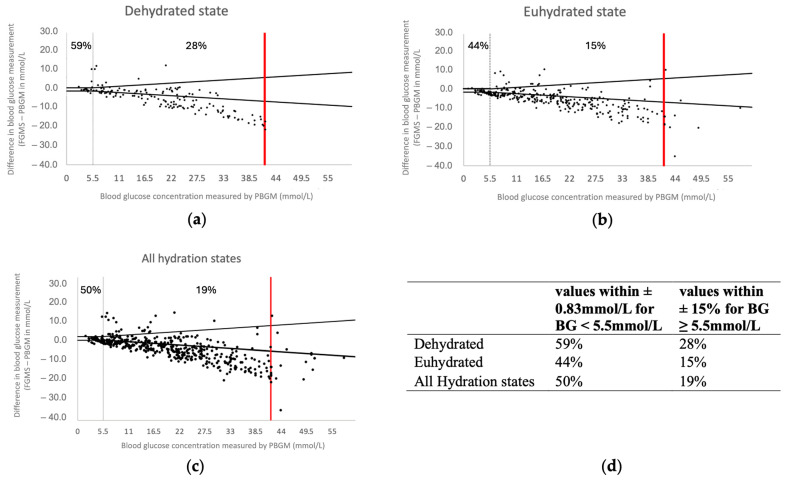
(**a**–**d**) Bland–Altman plots of the difference in glucose measurements between IG measured by the FGMS and BG measured by the PBGM in all samples irrespective of hydration state in (**a**) samples collected in dehydrated state and (**b**), in samples collected in euhydrated state (**c**). The BG concentration measured by the PBGM on the *x*-axis is plotted against the difference in glucose measurements (FGMS—PBGM) on the *y*-axis. Red lines perpendicular to the *x*-axis indicate the upper limit of the reliable measuring range of the PBGM, as specified by the manufacturer. (**d**) Summary of the percentage distributions within the required limits: ±0.83 mmol/L for BG < 5.5 mmol/L or ±15% for BG ≥ 5.5 mmol/L. Abbreviations: PBGM, peripheral blood glucose meter; FGMS, flash glucose monitoring system.

Based on the Bland–Altman analysis, when analysing all glucose concentrations irrespective of hydration, the percentage of values within ±0.83 mmol/L of the BG concentration for BG concentrations < 5.5 mmol/L and within ±15% of the BG concentration for BG concentrations ≥ 5.5 mmol/L was 50% and 19%, respectively (Figure 3a).

**Figure 4:**
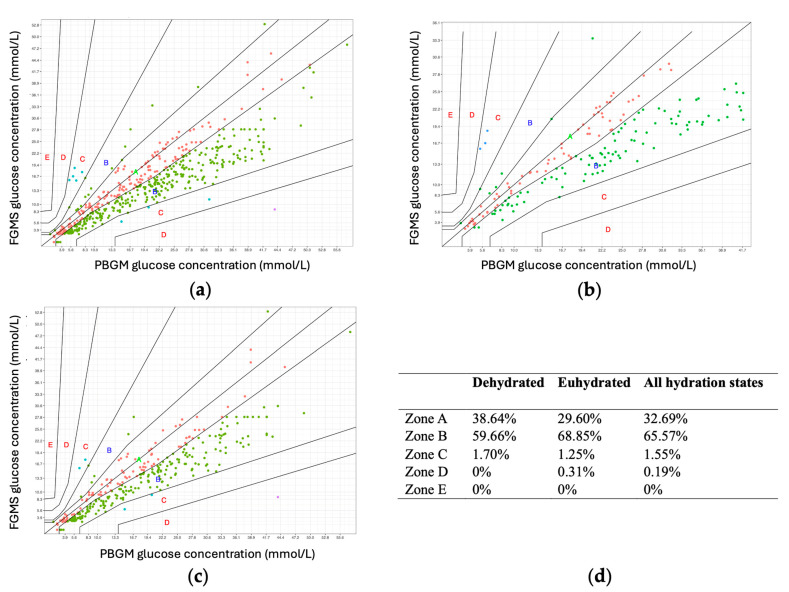
(**a**–**d**) Parkes consensus error grid analysis (EGA) representation with the percentage of values within different zones displaying (**a**) all measurements, (**b**) measurements from dehydrated cats only, and (**c**) measurements from euhydrated cats only. (**d**) Summary of percentage distributions within zones A–E. The reference glucose values (BG obtained by a PBGM) are plotted against the IG measurements obtained by the FGMS 2.0. The zones A–E represent the risk related to possible inaccuracies in measurements: no effect on clinical action (A), altered clinical action—little or no effect on clinical outcome (B), altered clinical action—likely to affect clinical outcome (C), altered clinical action—could have significant medical risk (D), altered clinical action—could have dangerous consequence (E). According to the ISO 15197:2013 guidelines, for a device to be considered accurate, it is required that 99% of the values fall within zones A+B. Abbreviations: EGA, error grid analysis; PBGM, peripheral blood glucose meter; FGMS, flash glucose monitoring system.

Parkes consensus error grid analysis (EGA) showed that 98.26% of the samples fell within zones A and B (508/517), 1.55% fell within zone C (8/517) and 0.19% within zone D (1/517) (Figure 4a).

### 3.5. Hydration State

A total of 176 values were recorded in clinically dehydrated state, and 321 values were recorded in clinically euhydrated state. Information on hydration status was not recorded for one cat (Figure 1, 20 measurements) and those values were excluded from further analysis regarding differences between hydration states.

IG concentration was significantly lower (*p* < 0.001) than BG concentration in both eu- and dehydrated cats, indicating that the FGMS 2.0 underestimated the glucose concentration compared to the PBGM (Table 1).

All accuracy measures were below 60% (Table 2). In the high glucose range, the accuracy was especially low, with only 15% and 28% of values within ±15% difference of the BG concentration in euhydrated or dehydrated states, respectively.

There was no significant association (KWA, *p* = 0.24) between hydration states and relative difference in the high glycaemic range (BG ≥ 5.5 mmol/L). Similarly, there was no significant association (KWA, *p* = 0.07) between hydration states and absolute difference in the low glycaemic range (BG < 5.5 mmol/L).

Analysis of the Bland–Altman plot performed separately for values obtained in eu- and dehydrated states showed similar results as the Bland–Altman plot using all values, suggesting that hydration status had no significant impact on the agreement between FGMS 2.0 and PBGM (Figure 3b,c). Glucose concentrations measured by the FGMS 2.0 were most often lower than glucose concentrations measured by the PBGM, with a few exceptions, which occurred mainly at mild to moderate hyperglycaemia (Figure 3a–c).

Analysis of Parkes EGA performed separately for values obtained in eu- and dehydrated states showed similar results as Parkes EGA using all values, suggesting that hydration status had no significant impact on the agreement between the FGMS 2.0 and PBGM (Figure 4b,c).

### 3.6. Inter-Individual Variability

**Figure 5:**
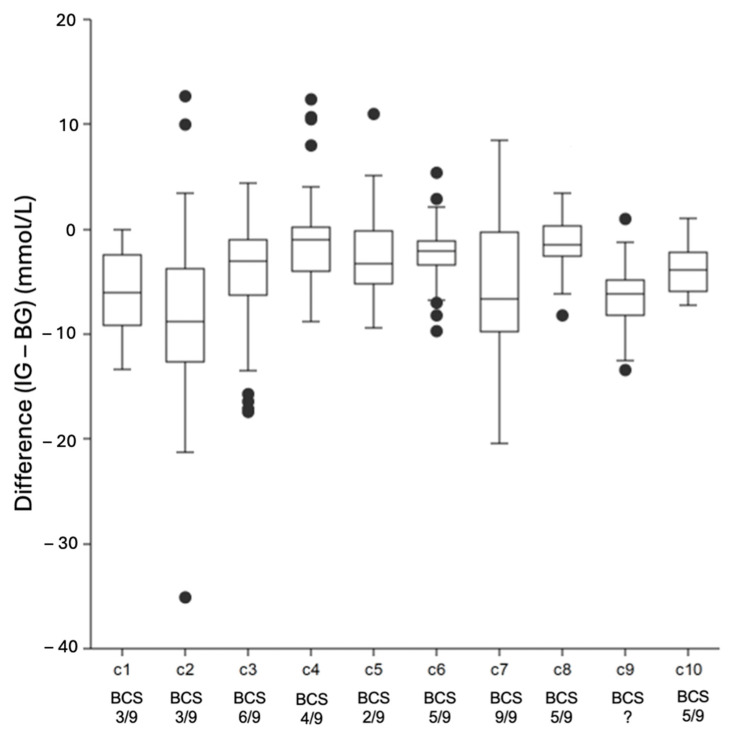
Box plots displaying inter-patient variability. Each box plot represents the differences between IG measured by the FGMS and BG measured by the PBGM of a single cat. Central lines within the boxes represent median values, whereas the borders of the box represent inter-quartile ranges. The whiskers indicate range outside the upper and lower quartiles, and the dots represent outliers. For each individual cat, body condition scores (BCS) are depicted where available. KWA was performed and revealed significant inter-patient differences between the FGMS 2.0 and PBGM measurements (*p* < 0.000001). Abbreviations: BG = blood glucose, IG = interstitial glucose, KWA = Kruskal–Wallis ANOVA.

Inter-individual variability in the difference between IG and BG concentrations is displayed in Figure 5. There was evidence of significant inter-patient differences in the agreement between the FGMS 2.0 and PBGM measurements (KWA, *p* < 0.000001). All median and third quartiles were below zero (zero indicating perfect accuracy), demonstrating that the FGMS 2.0 measurements were lower than the PBGM measurements. 

Table 3 depicts pairs of cats with significant differences after post-hoc Dunn-Bonferroni was performed. 

### 3.7. Owner Questionnaires

Five of ten questionnaires were returned to assess the feasibility of at-home IG monitoring via the FGMS 2.0. One cat was lost to follow-up after discharge, two cats died during hospitalisation and in two cats the sensor failed before discharge. According to the owners, the FGMS 2.0 was easy (3/5) or uncomplicated (2/5) to use. All owners (5/5) were able to scan the sensor without additional help or support. The tolerability of the sensor showed high individual differences ranging from the cat not being disturbed by the sensor at all (2/5) to being mildly (1/5) or moderately (1/5) disturbed, to being severely (1/5) bothered by the sensor. Three (60%) respondents reported that they would recommend the FGMS 2.0 to all owners of diabetic cats, and two (40%) only to some.

## 4. Discussion

This study is the first to evaluate the agreement between IG concentrations measured by the FGMS 2.0 (FreeStyle Libre Abbott^®^ 2.0) and blood glucose concentration measured by the PBGM in cats with DK(A). While there was a good overall correlation between the FGMS and PBGM, the FGMS 2.0 fulfilled neither analytical nor clinical accuracy criteria based on ISO 15197:2013 guidelines. Hydration status did not influence the accuracy of the FGMS.

Placement of the FGMS 2.0 was easy to perform. During hospitalisation, the sensor was well tolerated by the cats, which showed no signs of pain or other adverse reactions. The neck was chosen as the primary site of application, as it has been shown to produce the most accurate measurements in a previous study using a CGM (Guardian Real-Time, Medtronic) [20]. The author’s personal preference is to place the sensor in the dorsolateral neck area. With regards to fixation of the FGMS, several methods have been reported in the past, including no additional fixation, stitching or the use of cyanoacrylate tissue glue. Recent studies using the FGMS with or without additional glue have reported adverse dermatological reactions such as erythema, crust formation, skin erosions or abscessation at the site of application [4,13,17,18,26,32,33,34]. These complications were not noted in the present study, suggesting that the dermatologically tested human eyelash glue causes less dermatological irritation than cyanoacrylate tissue glue or skin stitches. None of the sensors was displaced during the functional period of the sensor. However, the functionality of the sensor was markedly shorter (median 5.5 days) than the maximum time indicated by the manufacturer (14 days). These results are comparable to previous reports using alternative attachment methods [4,13,17,18,26,32].

In this study, the results of the FGMS 2.0 measuring IG concentrations were compared to BG measurements via the PBGM. Overall, there was a good correlation between the two measuring methods and compartments. However, the FGMS 2.0 measured significantly lower glucose concentrations than the PBGM, which is consistent with some earlier studies in dogs and cats [4,6,14]. In contrast, one study involving stable diabetic dogs found that the median glucose concentration measured by the first-generation FGMS was higher than that measured by the PBGM [13].

In adult human diabetics, the FreeStyle Libre 2.0 has been shown to have a MARD of 8% when compared to a handheld glucometer, indicating good agreement [23]. This MARD is much lower than those reported in veterinary medicine [4,13,17,18,26,32,33,34]. Indeed, in the present study, the MARD between the FGMS 2.0 and the PBGM was above 30%. Although the manufacturer of the AlphaTRAK 2 does not report a MARD, it claims an average bias to reference of −1.6% and a coefficient of variation of 5.3% in cats [35]. We therefore assume that the high MARD between the FGMS 2.0 and PBGM is due to the FGMS 2.0’s lack of accuracy.

Previous studies have suggested utilizing criteria proposed in the ISO 15197:2013 guidelines to evaluate the usability of the FGMS in veterinary medicine [4,13,17,18,26,32,33,34]. The ISO 15197:2013 criteria, however, stem from human medicine and are designed to compare PBGMs with the standard reference method (hexokinase method) in stable diabetic patients. In veterinary medicine, there are no established accuracy criteria for a CGMS or the FGMS. While there is some disagreement among veterinary endocrinologists about whether the ISO 15197:2013 standards are appropriate for comparing interstitial glucose measurements from the FGMS to blood glucose measurements from a PBGM, the ISO 15197:2013 is still widely used. Whether the ISO 15197:2013 criteria are applicable to measurements in metabolically unstable patients and whether they can be extrapolated from human to veterinary medicine requires further study. Despite these limitations, the ISO 15197:2013 guidelines were used in this study to ensure comparability with other research.

In our study, the FGMS 2.0 did not meet either the analytical or clinical accuracy criteria based on ISO 15197:2013 guidelines. Clinical accuracy was evaluated using the Parkes EGA analysis. The total percentage of observations within acceptable limits (98.26%) was 0.74% below the desired limit of 99%. Recent studies in cats with uncomplicated DM have demonstrated sufficient clinical accuracy of the FGMS, showing good agreement between BG and IG [6,17,18,33]. However, during periods of rapid BG concentration changes, no correlation was found between BG and IG [6]. Similar results were observed in healthy cats receiving IV infusion of regular insulin to induce hyperinsulinaemic–hypoglycaemic clamps. In that study, BG and IG correlated strongly in stable glycaemia but only moderately during changes in glycaemia, which aligns with our findings [27]. Interstitial glucose in cats tends to change more slowly compared to blood glucose, with a delay that can last up to 30 min [6,7]. This delay may account for the reduced clinical accuracy of the FGMS 2.0 in this and other studies involving cats with DK(A), where significant BG fluctuations can occur during the initial phase of treatment. In contrast, studies involving dogs with DKA have shown that the FGMS and PBGM correlated well, and the FGMS did meet the criteria for clinical accuracy [4,8,14,26].

In contrast to clinical accuracy, the analytical accuracy criteria proposed by ISO 15197:2013 were not met in any of the previously published studies in veterinary medicine, whether involving stable diabetic patients or those with DKA [4,6,13,14,17,18,19,26].

In our study, glucose concentrations measured by the FGMS 2.0 were significantly lower than glucose concentrations measured by the PBGM. This might be partly caused by the delayed change of the IG concentration following rapid changes in the BG concentration, as discussed above. However, during rapid fluctuations in BG concentration, both over- and underestimation rather than underestimation would be expected. Because other studies in cats also identified some underestimation of glucose values by the FGMS [6,17,28], other causes such as manufacturer calibration should be considered. Underestimation of the glucose value in DKA patients might lead to inappropriate reduction of the insulin dose, temporary interruption of insulin treatment or prompt glucose supplementation, which might prolong the treatment period. On the other hand, dangerous hypoglycaemic events might be detected earlier or be prevented from happening, which in the clinical setting appears more imperative.

In the present study, hydration status of the cats did not affect the analytical or clinical accuracy of the FGMS 2.0. This contrasts with an earlier report on dogs with DKA [4]. The cause for this difference is uncertain. Factors such as skin thickness, position of the transcutaneous sensor (subcutis vs. cutis) or differences in the equilibrium mechanisms of IG might be contributing factors. The small sample size of 10 cats could also explain why a higher accuracy of the sensor in euhydrated patients was not detected.

Significant inter-individual variability in the difference between BG and IG measurements demonstrated in our study is in agreement with similar results in dogs with DKA [14]. The reasons for the inter-individual variability are unclear. In an older study evaluating another continuous glucose monitoring system (CGMS Gold, Medtronic Minimed, Northridge, CA, USA) in dogs and cats with DKA, only a weak association with hydration status seemed to be influential [6]. Another study evaluating the first-generation FGMS in dogs with DKA could not find any influential factors that could explain the inter-individual variability [14]. A possible influencing factor could be a different skin thickness in the individuals, as this has been shown to influence the clinical accuracy in diabetic dogs, with thicker skin leading to a better accuracy [36]. Skin thickness was not assessed in our study, as data collection was already underway at the time of publication of the aforementioned paper, and therefore no statement regarding the influence of the thickness of the skin can be made. In this cohort, both cats with high and low body condition scores showed high variability; thus, BCS seem unlikely to be a relevant factor.

Data from discharged cats indicate that owners found using the FGMS to be straightforward. No additional person was needed to help to acquire the IG measurements at home. In contrast, a study in 38 cats showed that owners required assistance of an extra person in 63% of the cases when using a PBGM for home blood glucose monitoring [37]. The inter-individual tolerance of the sensor by the cats was very variable and some cats sometimes tolerated the sensor well but were then disturbed when the sensor was renewed. While some cats were not affected by the sensor, some cats showed adverse behaviours towards the sensor. Anecdotal data provided by the Abbott Laboratories Customer Support in direct communication with the authors show that human patients also report diverging tolerance depending on the location of the sensor placement. This might be due to inadvertent injury to a subcutaneous nerve during sensor placement and continuous nerve irritation during application period.

Our study had several limitations. An inherent limitation of this study is the comparison of glucose concentrations measured using two different methods in two distinct compartments. With this study design, it is not possible to determine whether the observed differences are due to the limited accuracy of one method or to actual concentration differences between the two compartments or anatomical regions. Glucose concentrations measured by the FGMS 2.0 were compared to glucose concentrations measured by a PBGM validated for use in cats, instead of the classical hexokinase reference method. Using the hexokinase method in cats with naturally occurring DK(A), however, can lead to hospital-acquired anaemia because of frequent blood sampling [5]. To avoid these adverse effects, blood samples were only analysed by venous blood gas analysis (hexokinase) when additional information on electrolytes or acid-base-state was warranted. As samples were taken during hospitalisation and cats were discharged depending on the clinical state, the number of samples collected differed between cats. As there was a significant inter-individual difference in the accuracy of the FGMS, cats in which a higher number of samples were collected might have biased the results of the study. Lastly, the sample size and power in our study, although sufficient, were at the lower limit; thus, future studies with additional animals are required to confirm our results.

## 5. Conclusions

In conclusion, there was good overall correlation between the FGMS 2.0 and the PBGM. However, the FGMS 2.0 tended to measure lower interstitial glucose values than blood glucose levels as assessed by PBGM. Bland–Altman analysis and Parkes EGA indicated only limited clinical and analytical accuracy of the FGMS in cats with DK(A). While IG measured by the FGMS likely does not impose a risk of insulin overdosing, it may nonetheless lead to suboptimal insulin management. Additionally, a strong inter-individual variability in the difference between the two measurement techniques was noted. Although the FGMS 2.0 is well tolerated by cats and easy to use, currently this method cannot be recommended for routine monitoring of cats with DK(A). 

## Figures and Tables

**Table 1:** Comparison of blood and interstitial glucose concentrations in euhydrated and dehydrated cats (Wilcoxon signed-ranks Test). Abbreviations: BG = blood glucose, IG = interstitial glucose.

	Median BG Concentration (Range) mmol/L	Median IG Concentration (Range) mmol/L	*p*-Value
Dehydrated state	20.9 (2.6–41.8)	14.8 (2.9–33.6)	<0.001
Euhydrated state	16.7 (1.7–57.5)	13 (1.1–53)	<0.001

**Table 2:** Analytical accuracy of the FGMS in the low (BG < 5.5 mmol/L) and high glycaemic range (BG ≥ 5.5 mmol/L) in euhydrated and dehydrated cats. Abbreviations: n, number of measurements; BG, blood glucose; MAD, mean absolute difference; MARD, mean absolute relative difference; mARD, median absolute relative difference; MRD, mean relative difference.

	Dehydrated	Euhydrated
**Low glucose range (<5.5 mmol/L)**		
n	17	34
MAD (mmol/L)	1.41	1.06
% of values within ±0.83 mmol/L of the BG value	59% (10/17)	44% (15/34)
**High glucose range (≥5.5mmol/L)**		
n	159	287
MARD (%)	38.1%	44.5%
mARD (%)	35%	39.8%
MARD (%)	−35.8%	−41.3%
% of values within ± 15% of the BG value	28% (44/159)	15% (43/287)

**Table 3:** Post hoc Dunn–Bonferroni analysis was performed to identify pairs of cats with statistically significant differences. Only significant comparisons are included; non-significant comparisons are omitted.

Comparison between Cats	*p*-Value after Bonferroni Correction
c2 and c3	<0.001
c2 and c5	<0.001
c3 and c8	<0.001
c3 and c10	<0.001
c4 and c5	0.001
c5 and c7	<0.001
c5 and c8	<0.001
c5 and c10	<0.001

## Data Availability

The original contributions presented in the study are included in the article/Supplementary Materials, further inquiries can be directed to the corresponding author.

## Supplementary Materials

The following supporting information can be downloaded at https://www.mdpi.com/article/10.3390/ani14192787/s1, Document S1: Quality of life questionnaire for at-home use of the FGMS 2.0.

## Abbreviations

BG (blood glucose), BCS (body condition score), CGM (continuous glucose monitoring), CRI (continuous rate infusion), DK(A) (diabetic keto(acido)sis), DKA (diabetic ketoacidosis), EGA (error grid analysis), FGMS (flash glucose monitoring system), IG (interstitial glucose), IQR (interquartile range), KWA (Kruskal–Wallis ANOVA), MAD (mean absolute difference), MARD (mean absolute relative difference), mARD (median absolute relative difference), MRD (mean relative difference), PBGM (portable blood glucose meter), SC (subcutaneous), SD (standard deviation).
