# Supplementary material for: Comparison between a Flash Glucose Monitoring System and a Portable Blood Glucose Meter for Monitoring of Cats with Diabetic Ketosis or Ketoacidosis
Source: Animals (Basel). 2024 Sep 26;14(19):2787. doi: 10.3390/ani14192787 (PMC11475600; doi:10.3390/ani14192787)
Supplement: Supplementary file 1 [file animals-14-02787-s001.zip › animals-3168467-supplementary.pdf]

Was the initial introduction to FreeStyle Libre in our clinic and the subsequent support and advice sufficient?
o Yes, I felt well-prepared after the introduction, and most questions were adequately answered.
o Yes, but a significant number of questions were not adequately answered.
o No, but I received enough support afterwards.
o No, and I did not receive sufficient support afterwards.

How easy/difficult is it for you to perform a blood glucose measurement at home with the FreeStyle Libre 2.0?
o Difficult
o Mostly uncomplicated[1]
o Easy[2]

What was the most challenging part of the blood glucose measurement for you?
o Using the FreeStyleLibre app.
o Holding my cat.
o Finding time to perform the measurements.
o Gaining confidence in doing this.
o Other (please describe):

How well did your cat tolerate the FreeStyle Sensor at home?
o My cat didn't seem bothered by the sensor.
o My cat seemed slightly bothered by the sensor and occasionally scratched on it.
o My cat seemed moderately bothered by the sensor/dressing and was partially restricted in movement or frequently scratched on it.
o My cat seemed highly bothered by the sensor/dressing and immediately removed the sensor or was significantly affected in her quality of life.

Do you feel that your life is restricted by performing blood glucose measurements at home?
o Yes, very restricted.
o Yes, moderately restricted.
o Yes, a little restricted.
o No, not really.
o No, not at all.

What are the benefits of performing blood glucose measurements at home for you? Please tick as many options as applicable to you and your cat.
o I feel like I have more control over my cat's diabetes condition.
o I feel that my cat's diabetes is better controlled since I started performing blood glucose measurements at home.
o I don't have to take my cat to the vet as often.
o I don't have to leave my cat at the vet's practice as frequently.
o My cat seems less stressed compared to vet visits when she undergoes monitoring at home.
o Conducting measurements at home is cheaper for me.
o Other (please describe):

How satisfied are you with FreeStyle Libre 2.0?
o I am very satisfied with FreeStyle Libre 2.0, and I found blood glucose measurement easy.
o I am quite satisfied with FreeStyle Libre 2.0, and I found blood glucose measurement mostly manageable.
o I am rather dissatisfied with FreeStyle Libre 2.0, and I found blood glucose measurement mostly complicated.
o I am not satisfied with FreeStyle Libre 2.0, and I found blood glucose measurement difficult.

Would you recommend home blood glucose monitoring with FreeStyle Libre to a friend with a diabetic cat?
o Yes, always.[3]
o For some, but not others.[4]
o No, never.

Do you need another person to help you with blood glucose measurements (e.g., to hold your cat, assist with the measurement, etc.)?
o Yes, always.
o Sometimes.
o No, never.

Was recording the data on the FreeStyle Libre software clear to you?
o Recording the data using the software was straightforward and reliable.
o Recording the data using the software was mostly uncomplicated and reliable.
o Recording the data using the software was frequently complicated or unreliable.
o Recording the data using the software was very complicated or unreliable.

Please feel free to provide suggestions on how the introduction to home blood glucose monitoring can be improved, or share your positive/negative experiences with home blood glucose monitoring. Also, describe any specific issues you have encountered or any advice you would give to owners (or veterinarians) of diabetic cats conducting home monitoring.

---

[1] The sensor was working smoothly most of the time, but occasionally some problems arose.
[2] The sensor worked smoothly all of the time, and no problems arose.
[3] I believe the sensor is well suited for people of all ages and I would recommend it in any case.
[4] I believe the sensor is well suited for most people, but some people might find the sensor hard to handle or is too expensive.
